# Physiological significance of vitamin D produced in skin compared with oral vitamin D

**DOI:** 10.1017/jns.2022.11

**Published:** 2022-02-21

**Authors:** David R. Fraser

**Affiliations:** Sydney School of Veterinary Science, Faculty of Science, The University of Sydney, RMC Gunn Building (B19), Sydney, NSW 2006, Australia

**Keywords:** 25-hydroxyvitamin D production, 25-hydroxyvitamin D toxicity, Angiotoxicity, Vitamin D transport, 7-DHC, 7-dehydrocholesterol, 25(OH)D, 25-hydroxy vitamin D, DBP, vitamin D-specific-binding protein

## Abstract

Since the discovery of vitamin D, it has been accepted that its physiological supply is either from food or by endogenous synthesis in skin exposed to solar UV light. Yet vitamin D is a component of very few foods and its supply as a natural nutrient is unable to maintain good vitamin D status for human populations. One aspect of vitamin D physiology that has been ignored is that the mechanisms for its transport and processing from these two sources are quite different. Excess intake of vitamin D causes hypercalcaemic toxicity. However, experiments with different animal species have shown that long-term supply of oral vitamin D in apparently non-toxic amounts causes atherosclerosis in large arteries. A mechanism for this toxicity is proposed. Alternative strategies for addressing widespread vitamin D deficiency by food fortification should be considered in light of the angiotoxicity caused by oral vitamin D in animal experiments.

Since the discovery, more than 100 years ago, of the small molecules known as organic micronutrients or vitamins, it has been axiomatic that their physiological source is from food and that they enter the body by absorption from the alimentary tract. The explanation as to why these essential enzyme co-factors or cell regulating substances can be made by plants and bacteria and not by terrestrial animals is that evolutionary efficiency eliminates the metabolic synthesis of substances that can be reliably obtained from food. A specific example is the genetic loss of the enzyme L-gulono-gamma-lactone oxidase in primates and a few other vertebrate families^(1)^. This enzyme catalyses the final step in the biosynthesis of ascorbic acid (vitamin C). Its genetic loss removed a dispensable metabolic process from those species which had reliable dietary sources of vitamin C.

However, one of these organic micronutrients, vitamin D, can be synthesised in humans and most other terrestrial vertebrates, but not by an enzyme-catalysed metabolic process. Rather, vitamin D is produced in skin as a photochemical product from the action of UV light on its precursor, 7-dehydrocholesterol (7-DHC), when the skin is exposed to the sun^(2)^. Because vitamin D was discovered as a nutritional factor that prevented and healed the bone disease of rickets in dogs^(3)^, it has become accepted dogma that vitamin D supply can be either from food or by formation in skin^(4)^. With the discovery of the secosteroid structure of vitamin D and the ability for its commercial production as either cholecalciferol from 7-DHC or as ergocalciferol from the fungal and yeast sterol, ergosterol, food was able to be fortified to increase artificially the contribution of oral vitamin D to vitamin D status. Nevertheless, despite food fortification, it is apparent from the seasonal changes in vitamin D status of populations that the main supply of vitamin D is from its photochemical synthesis in skin by the action of solar UVB light^(5)^.

The natural foods that contain enough vitamin D to contribute to vitamin D status are fish, meat, milk, and eggs^(6,7)^. Hence, these would have been the nutritional sources of vitamin D before food fortification or oral supplements were available. It is likely that the vitamin D content of hen eggs is higher than before the discovery of vitamin D, as the diet of high-producing egg-laying hens is now fortified with vitamin D to maintain egg-shell quality. Recent analyses of the amounts of vitamin D_2_, vitamin D_3_ and their 25-hydroxy metabolites in these foods of animal origin are summarised in Table 1. The oral intake of 25-hydroxy vitamin D [25(OH)D] has been shown to be up to five times more effective than the parent molecule at raising the concentration of 25(OH)D in blood serum^(8)^. Therefore, a diet with varying amounts of these food components would supply up to 15 μg of vitamin D equivalents per day. Dietary surveys of populations from around the world indicate, however, that the typical range of vitamin D daily intakes from unfortified food is from 0 to 5 μg/d^(9)^. It would follow that this would have been the range of daily intakes of vitamin D in human history before the discovery of this hormone precursor. Recommended daily intakes of vitamin D range from 10 to 15 μg^(10)^, to maintain adequate vitamin D status, often defined as a serum 25(OH)D concentration of >50 nm.

**Table 1.** Vitamin D content of unfortified food

	Vitamin D_3_		25(OH)D_3_		Vitamin D_2_		25(OH)D_2_	
Reference No.	(6)	(7)	(6)	(7)	(6)	(7)	(6)	(7)
Milk^a^ (μg/l)	0·4	0·08	0·12	0·04	0·12	−	−	−
Egg (μg/egg)	0·55	0·72	0·3	0·24	−	−	−	−
Beef (μg/100 g)	0·12	0·06	0·17	0·16	0·17	0·027	0·06	0·063
Salmon (μg/100 g)	8·7	8·7	0·18	0·18	−	−	−	−

a Full-cream milk.

How does this recommendation compare with the potential quantity of vitamin D produced by skin exposure to the sun? There are many variables that affect the efficiency of conversion of 7-DHC to vitamin D_3_. The most obvious is the seasonal variation in the intensity of solar UVB radiation, which accounts for the higher vitamin D status of populations in summer compared with winter^(11)^. Other variables include the decline in 7-DHC content of skin with age^(12)^, the proportion of body surface area exposed to the sun, the absorption of UVB radiation by skin pigmentation^(13)^ and the conversion of pre-vitamin D_3_ to inactive products, lumisterol and tachysterol, with prolonged exposure to UV radiation^(14)^. Bearing in mind these variables, the potential yield of vitamin D_3_ can still be estimated using modest values of 7-DHC μg/cm^2^, percentages of 7-DHC converted and percentages of the total skin area^(15)^ exposed (Table 2). It can be seen that not only is the likely quantity of vitamin D formed in skin considerably greater than the likely intake from non-fortified food, but it could also reach values regarded as potentially toxic, if such quantities were to be ingested^(17)^. Yet even with a high proportion of the total skin area being exposed for many days in summer, there is no evidence that the accumulating supply of vitamin D_3_ causes hypervitaminosis D toxicity.

**Table 2.** Potential yield of vitamin D_3_ formed in skin from a single exposure to UV sunlight

	5 % skin^a^ exposed (μg)	10 % skin^a^ exposed (μg)	20 % skin^a^ exposed (μg)
7-DHC^b^ at 0·5 μg/cm^2^	458	917	1834
Vitamin D_3_ produced at 10 % conversion	46	92	183
Vitamin D_3_ produced at 20 % conversion	92	183	367

The yield of vitamin D_3_ in skin, in response to exposure to UV light from the sun, depends not only on the number of minutes of a single period of exposure, but also on many other variables, including the seasonal intensity of solar UVB radiation, absorption of UVB radiation by skin pigmentation, the age of the irradiated individual and the proportion of pre-vitamin D_3_ produced that is then converted by prolonged irradiation, to other non-biologically active products. Experiments with human skin, irradiated *in vitro*, have found up to 35 % of total 7-DHC that can be converted to pre-vitamin D_3_ in a single exposure session^(16)^.

a Based on the calculated total skin area of an adult male of 18 229 cm^2(15)^.

b Lowest concentration of 7-DHC in skin, which ranges from 0·5–1·3 μg/cm^2(12)^.

Because vitamin D has been regarded as a micronutrient, it has been assumed that its supply from food and from its formation in skin were physiologically equivalent. But there are differences in their transport and rate of conversion to 25(OH)D. Pre-vitamin D_3_ produced in skin undergoes heat stimulated isomerisation to vitamin D_3_ at a rate partly determined by the skin temperature and partly by the presence of the vitamin D-specific-binding protein (DBP) in the fluid surrounding the keratinocytes in which the pre-vitamin D_3_ was produced. The high concentration of apo-DBP molecules in blood and extracellular fluid, each with a single binding site for vitamin D or its metabolites, enhances the rate of conversion of pre-vitamin D_3_ to vitamin D_3_ by tightly binding the vitamin D_3_ that diffuses from the skin cells, thus promoting the forward direction of this reversible isomerisation reaction^(18,19)^. This skin-derived vitamin D is then taken up by hepatic cells, a process that continues steadily for up to 7 d after a single exposure of skin to solar UV light^(20)^. In the liver cells, the vitamin D_3_ is 25-hydroxylated by the microsomal enzyme CYP2R1^(21)^ and the 25(OH)D_3_ then trickles into blood, induced to emerge from cells by the high-affinity, specific-binding sites of the plentiful apo-DBP in the circulation. Again, this accumulation of 25(OH)D_3_ in blood occurs slowly, taking between 7 and 14 d before the highest concentration is reached after a single exposure of skin to UV radiation^(22)^.

Now, contrast this with the fate of orally delivered vitamin D, where the mechanism and rate of delivery to the liver are quite different from those of vitamin D formed in skin. Dietary vitamin D is absorbed into intestinal mucosal cells in association with dietary fat^(23)^. This vitamin D, along with triacylglycerols, phospholipids and cholesterol, then traverses the baso-lateral mucosal cell membrane in chylomicron lipid particles. This chylomicron lipid in the circulation is then incorporated as a bolus into hepatocytes, with up to 50 % of the absorbed vitamin D taken up by those cells, in 1 h after entering the circulation^(24,25)^. Within hepatocytes, two metabolic processes occur simultaneously. One is the 25-hydroxylation of the orally supplied vitamin D. The other is the processing of the chylomicron lipids into triglyceride- and cholesterol-containing, very-low-density lipoproteins (VLDL)^(26)^. The 25(OH)D and VLDL products of these metabolic processes then exit the liver cells over the same time period, with the 25(OH)D being immediately bound to the apo-DBP in blood. However, because of the conformational similarity of vitamin D to cholesterol^(27)^, some of the 25(OH)D derived from oral vitamin D could become incorporated into the VLDL emerging from the liver cells.

The classical sign of vitamin D toxicity is hypercalcaemia, which occurs with persistent oral consumption of 250 μg/d or more^(17)^. The mechanism of this hypercalcaemic toxicity is attributed to an uncontrolled delivery of the vitamin D hormone, 1,25-dihydroxy vitamin D [1,25(OH)_2_D] to various cells which then promote excess calcium delivery into blood from both intestinal absorption and bone mineral mobilisation^(17)^. The reason suggested for why 1,25(OH)_2_D is able to enter cells at random is because more of it becomes “free” when high concentrations of 25(OH)D occupy much of the specific binding sites on DBP in blood^(17)^. The high concentration of 25(OH)D in blood has not itself been regarded as the mediator of the toxicity. Yet large doses of oral vitamin D are used as a rodenticide and are able to kill rats and mice in as short a time as 1 d after oral ingestion^(28)^, when very high concentrations of 25(OH)D are found in blood^(29)^.

An alternative hypothesis is that vitamin D toxicity is mediated not only by 1,25(OH)_2_D but rather by its far more plentiful precursor, 25(OH)D. At good vitamin D status of 50–80 nm 25(OH)D in blood serum and with a normal DBP concentration of 4–8 μm, only about 0·03 % of that 25(OH)D would not be tightly protein-bound^(30)^. Any of the unbound 25(OH)D that diffuses into cells, where there is no specific function for that metabolite, would quickly be induced to leave by the high concentration of apo-DBP in the extracellular environment. However, when 25(OH)D was added to aortic endothelial cell cultures, it became apparent that this vitamin D metabolite is quite cytotoxic, even at low concentrations (Fig. 1)^(31)^. It could thus be concluded that the very high concentration of apo-DBP in blood, relative to those of vitamin D and its metabolites, is an effective way of preventing the uncontrolled entry of 25(OH)D into cells.

**Fig. 1.**
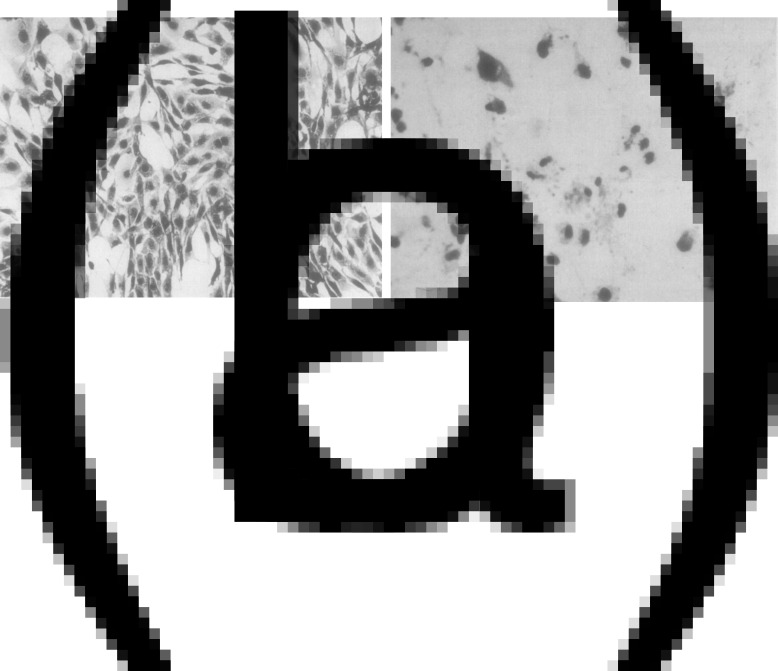
Pig aortic endothelial cells grown in culture: (a) control cells and (b) cells exposed for 24 h to 300 nm 25(OH)D_3_. Reproduced from Levene and Lawson^(31)^.

The toxicity of orally supplied vitamin D, independent of hypercalcaemia, has been demonstrated in several early studies in pigs, rabbits, rats and non-human primates. Angiotoxicity was the typical pathology found when vitamin D was fed in amounts that were greater than those needed to prevent deficiency, but considerably less than intakes causing hypercalcaemic toxicity. As an example of this arterial pathology, squirrel monkeys were fed daily doses of vitamin D that were five times higher than the amount required to maintain good vitamin D status^(32)^. The proliferation of myointimal cells and atheromatous plaques were found in the aorta of these animals (Fig. 2), but there was no hypercalcaemia nor calcium deposits in the arterial lesions. A yet unproven mechanism for the angiotoxicity in animal experiments, following repeated oral intake of vitamin D, could be the toxic action of 25(OH)D trapped in VLDL, when delivered to the endothelial and smooth muscle cells of arteries.

**Fig. 2.**
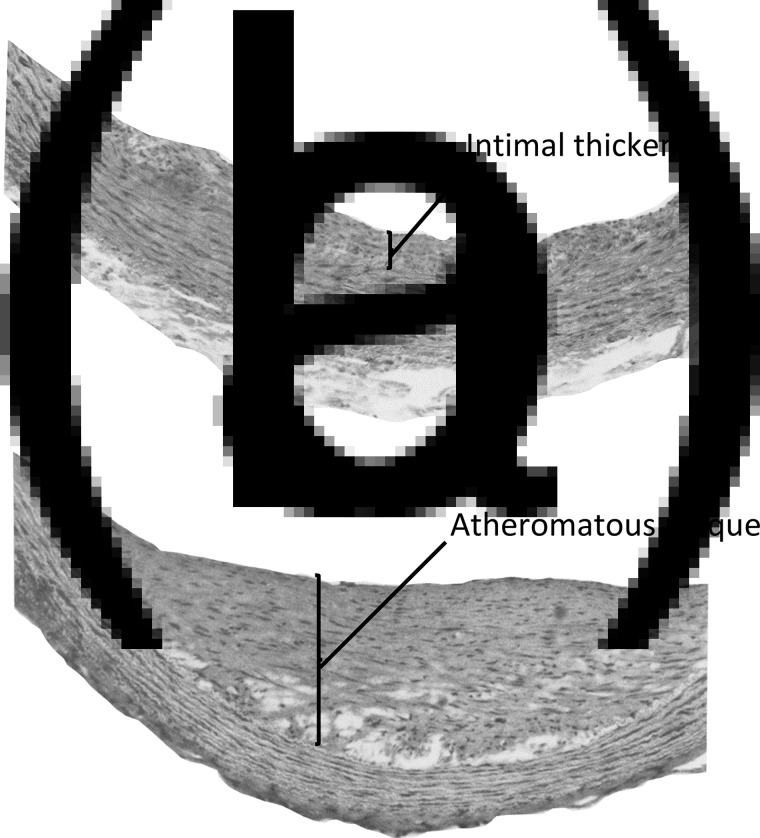
Aortic lesions in squirrel monkeys given daily oral doses of vitamin D. Adult squirrel monkeys weighing 750–1000 g were given daily oral doses of 12·5 μg vitamin D_3_ for 10–18 months with diets containing 0·5 % cholesterol. Histological sections of the aorta of these animals showed (a) intimal thickening with proliferation of myointimal cells and (b) atheromatous plaques. No aortic lesions were reported in animals on control diets containing 0·5 % cholesterol and providing 2·5 μg vitamin D_3_ per day. Reproduced with permission from Peng *et al.* ^(32)^.

The physiological supply of vitamin D by UV-induced formation in skin has evolved with transport and metabolism mechanisms that protect against the potential toxicity of 25(OH)D, regardless of the amount of vitamin D being produced. Another essential and potentially toxic lipophilic substance, retinol (vitamin A), has also to be obtained from the environment. But, in comparison to vitamin D, the vitamin A precursors, the carotenoids, are plentifully available in vegetarian foods. However, unlike orally supplied vitamin D, the mechanisms for metabolism and transport of vitamin A from food have evolved to protect against its potential toxicity^(33)^. The esterification of vitamin A with long-chain fatty acids, its complex storage process in hepatic stellate cells and its rigorously controlled release from the liver bound to the retinol-binding protein enable it to be safely delivered to the many cells where it performs regulatory and structural functions^(33)^.

The processing of vitamin D produced in skin has evolved in a way that protects against potential toxicity. In contrast, the maintenance of adequate status by the continuous oral supply of vitamin D has only been a strategy for the last 100 years. Thus, no protective adaptation against any vitamin D toxicity by this route, unlike the physiological processing of vitamin A, has had time to evolve. Animal studies demonstrate that long-term maintenance of vitamin D status by supplying oral vitamin D may be associated with vascular pathology. It would therefore be wise to develop population strategies to avoid this potential vitamin D toxicity, which utilise the physiology of vitamin D formation in skin. One such strategy could be the controlled exposure of skin to UV light in the vitamin D-producing wavelength range of 290–320 nm. However, the logistic of arranging this on a population basis, while preventing UV damage to skin cells, is a formidable challenge. An alternative method of obtaining vitamin D could be via transdermal administration, which would use the physiological transport and metabolism processes that have evolved to ensure a safe supply of this potentially toxic substance.
